# Bacterial symbionts influence host susceptibility to fenitrothion and imidacloprid in the obligate hematophagous bed bug, *Cimex hemipterus*

**DOI:** 10.1038/s41598-022-09015-0

**Published:** 2022-03-22

**Authors:** Li-Shen Soh, G. Veera Singham

**Affiliations:** Centre for Chemical Biology, Universiti Sains Malaysia, 11900 Bayan Lepas, Penang Malaysia

**Keywords:** Molecular biology, Physiology, Microbiology, Bacteria, Microbial communities

## Abstract

The use of insecticides remains important in managing pest insects. Over the years, many insects manifested physiological and behavioral modifications resulting in reduced efficacy of insecticides targeted against them. Emerging evidence suggests that bacterial symbionts could modulate susceptibility of host insects against insecticides. Here, we explore the influence of host microbiota in affecting the susceptibility of insect host against different insecticides in the blood-sucking bed bug, *Cimex hemipterus*. Rifampicin antibiotic treatment resulted in increased susceptibility to fenitrothion and imidacloprid, but not against deltamethrin. Meanwhile, the host fitness parameters measured in the present study were not significantly affected by rifampicin treatment, suggesting the role of bacterial symbionts influencing susceptibility against the insecticides. 16S metagenomics sequencing revealed a drastic shift in the composition of several bacterial taxa following rifampicin treatment. The highly abundant Alphaproteobacteria (*Wolbachia* > 90%) and Gammaproteobacteria (*Yersinia* > 6%) in control bed bugs were significantly suppressed and replaced by Actinobacteria, Bacilli, and Betaproteobacteria in the rifampicin treated F1 bed bugs, suggesting possibilities of *Wolbachia* mediating insecticide susceptibility in *C. hemipterus*. However, no significant changes in the total esterase, GST, and P450 activities were observed following rifampicin treatment, indicating yet unknown bacterial mechanisms explaining the observed phenomena. Re-inoculation of microbial content from control individuals regained the tolerance of rifampicin treated bed bugs to imidacloprid and fenitrothion. This study provides a foundation for a symbiont-mediated mechanism in influencing insecticide susceptibility that was previously unknown to bed bugs.

## Introduction

Insects are long-known to have established various relationships with symbiotic bacteria, whereby the insect host depends on the bacteria for sustaining different life aspects such as protection against pathogens^1^ and parasitism^2^, nutrition provisioning^3^, and modulating host behaviour^4^. This relationship enables host’s adaptive radiation into a new niche that would be inaccessible without the symbiont^5,6^.

The pest arthropods from several insect families including Culicidae, Cimicidae, Pediculidae, and Pthiridae feed on vertebrate blood to support their growth and development^7,8^. Nonetheless, the availability of essential nutrients such as vitamins and amino acids are often limited in their blood meals^9,10^. The symbiotic association between the blood-sucking insects and microbes helps in overcoming these restraints, as the microbes proffer the nutrients which are necessary for the insect host survival^9,10^. This mutualistic association which benefits both the host and symbionts has been well-documented in various blood-sucking insects and other arthropods especially B vitamins provisioning in tsetse flies^11^, human lice^12–14^, bed bugs^3,15^, and ticks^16^ by the bacterial symbionts *Wigglesworthia glossinidia*, Candidatus *Riesia pediculicola*, *Wolbachia* sp., and *Francisella* F-Om, respectively.

Bed bugs (*Cimex lectularius* and *Cimex hemipterus*) are obligate anthropophilic blood-sucking ectoparasites that have now emerged as important public health pests globally. The former species is commonly found in temperate regions whereas the latter can be found mainly in the tropical and subtropical regions^17,18^. Bed bug bites can cause several physical and medical complications from mild to severe allergic reactions such as erythematous rash, lesion, papules or wheals, and hemorrhagic bullae, as well as psychological impact like anxiety, and insomnia^19,20^. Bed bugs were once almost completely eradicated from developed nations in the late 1940s mainly due to widespread and liberal use of DDT and chlorinated hydrocarbon pesticides^21^. Nonetheless, bed bugs infestations have resurged globally in the last two decades largely due to insecticide resistance and increased rate of international travel which facilitated their spread globally^18,22,23^.

Chemical control based on the application of residual insecticides remains the primary choice for managing bed bugs infestations^19,21^. Numerous studies have reported insecticide resistance in bed bugs to most major insecticide classes including pyrethroid^24–27^, organophosphate^28–31^, neonicotinoid^32^, carbamate^31^, chlorinated hydrocarbons^33,34^, and phenylpyrazole^35^. Several mechanisms have been identified to explain the reduced efficiency of insecticides in bed bugs which include penetration resistance due to enhanced cuticle thickness^36^, metabolic resistance due to enhanced expression of xenobiotic metabolizing enzymes^29^, and reduced target site sensitivity due to mutations that causes binding inefficiency of pyrethroids in the voltage gated sodium channels^24,25,37^.

Given the long evolutionary history between bacterial symbionts and insects, it is probable that the microbes could play a significant role in mediating tolerance against xenobiotics to promote survival in insects. Although, it is widely known that various environmental microorganisms play a key role in the degradation of xenobiotics from the environment, their direct association in mediating insecticide susceptibility in insects was poorly understood or mostly ignored until the last few decades^38^. The work of Kikuchi et al.^39^ provided direct evidence of bacterial symbiont mediating susceptibility to insecticide in the association between bean bugs (*Riptortus pedestris*) and fenitrothion degrading *Burkholderia* symbiont. Since then, there is a growing number of scientific reports that suggests increased tolerance of several pest insects to xenobiotics can be due to microbiome organisms and not due to mechanism within the genome of pest^38,40^. Therefore, we hypothesized that the bacterial symbionts in insects with highly restricted diet such as in the obligate blood-sucking insects likely influence the susceptibility of the host insect against xenobiotics to enhance their chances of survival in the pesticide contaminated environment.

Here, we propose that bacterial symbionts of the tropical bed bug, *C. hemipterus* can contribute to their susceptibility against several commonly used insecticides. To test our hypothesis, we subjected the bed bugs to antibiotic treatment and evaluated its impact against three major classes of insecticides including pyrethroid (deltamethrin), organophosphate (fenitrothion), and neonicotinoid (imidacloprid). To ensure that the antibiotic treatment did not disrupt host fitness, we supplemented the blood meal diet with B vitamins and observed egg hatchability, fecundity, and nymphal development. Metagenomics sequencing of 16S rRNA v3-v4 hypervariable regions were used to profile the changes in bacterial microbiota assemblage that influences susceptibility of the host bed bugs against the insecticides. We also investigated the enzymatic activities of xenobiotic metabolizing enzymes from the whole-body lysate of the bed bugs to gauge changes in metabolic activities upon disruption of host’s bacterial microbiota. Lastly, we conducted a transplanting experiment by transferring the microbiota from control individuals to antibiotic-treated bed bugs and observe if the tolerance to insecticides can be regained.

## Results

### The effect of antibiotic treatment on fecundity and nymphal development of C. hemipterus

**Figure 1:**
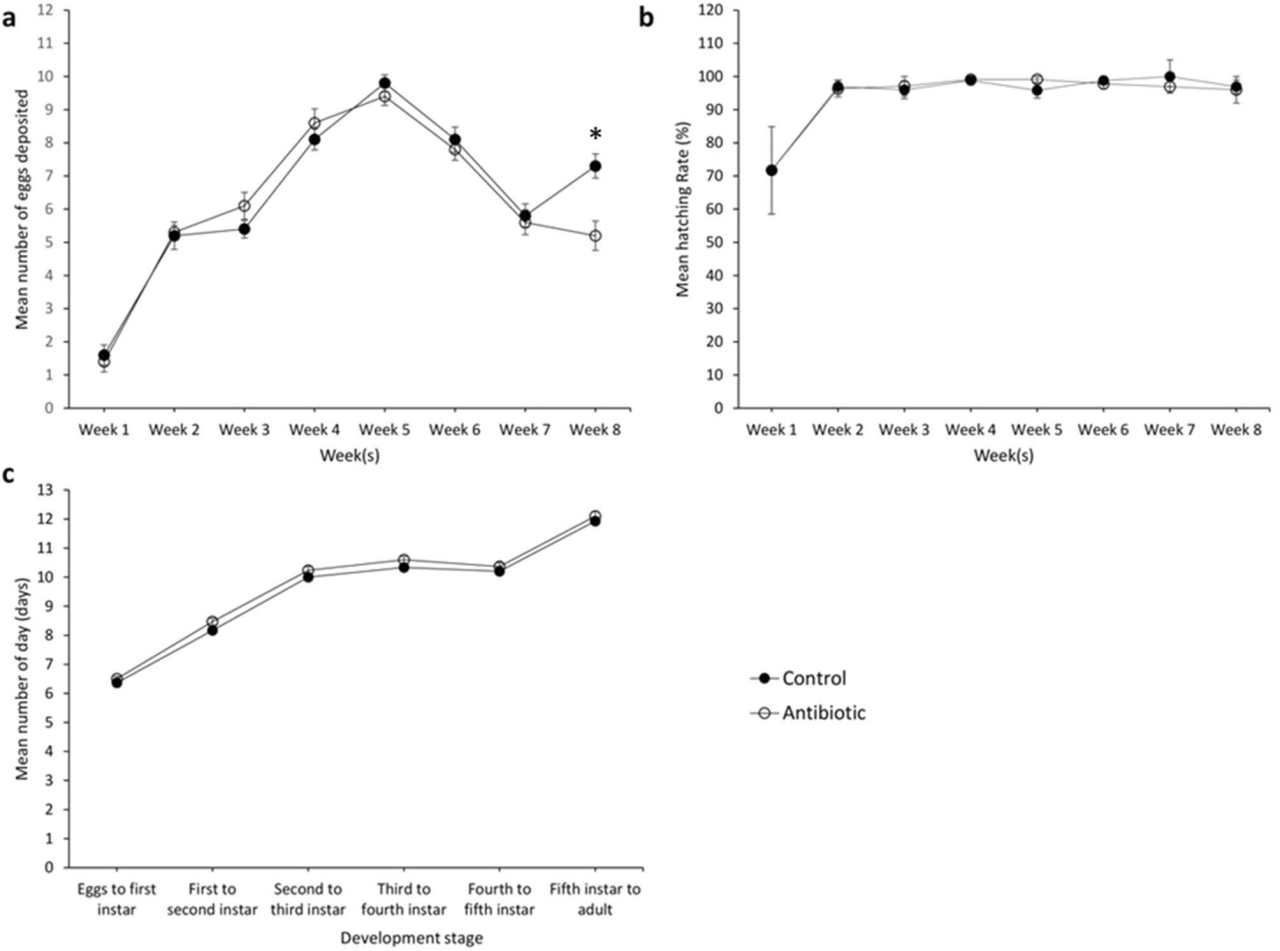
Effects of antibiotic treatment on different life history traits of *C. hemipterus*. (**a**) fecundity; (**b**) hatching rate; (**c**) nymphal development. Asterisk denotes significant difference, *P* < 0.05 (independent t-test).

The number of eggs deposited by the control and rifampicin-treated bed bugs was recorded on a weekly basis for up to 8 weeks (Fig. 1a). The results showed no significant differences in the total number of eggs deposited between the treatment and control groups during the first seven weeks, although a significant difference (two-sample independent t-test: P = 0.002, t = 3.656, df = 18) was observed at week 8 (Fig. 1a). Oviposition was most active during week 5 for both groups (Fig. 1a). Similarly, the intake of rifampicin-supplemented blood meal displayed no significant effect on the egg’s hatchability in the treatment group when compared with the untreated control throughout the evaluation period (Fig. 1b). Rifampicin treatment also revealed no significant effect in the developmental time of nymphs from eggs to adults when compared with untreated control (Fig. 1c). The mean number of days needed for the bed bugs to develop from eggs to adults was 57.0 ± 0.29 and 58.3 ± 0.28 days, for the control and treatment group, respectively.

### Insecticide bioassays of rifampicin-treated and untreated C. hemipterus

The effect of antibiotic treatment on the survivorship of bed bugs against insecticides was tested by subjecting the bed bugs to deltamethrin, fenitrothion, and imidacloprid. The survivorship of bed bugs in the rifampicin treated F0 and F1 groups was compared with the untreated control. Both F0 and F1 bed bugs displayed no significant difference in their survivorship against deltamethrin when compared with the control bed bugs; restricted mean survival time (RMST) were found to be > 1440 min (Table 1; Supplementary Fig. S1). Similarly, no mortality was observed 48 h post-treatment in all three groups (Table 1). On the other hand, rifampicin treatment resulted in significant differences in the survivorship of control, F0, and F1 bed bugs for fenitrothion and imidacloprid bioassays (see Supplementary Fig. S1). Based on the RMST values, bed bugs from the F0 and F1 groups were found to be significantly more susceptible to fenitrothion and imidacloprid when compared with the control (Table 1). Meanwhile, no significant difference in RMST values was observed between F0 and F1 bed bugs when tested against fenitrothion and imidacloprid (Table 1). Significant difference in time to knockdown 100% of bed bugs (KT100) was also found significantly faster in rifampicin treated F0 and F1 bed bugs when compared with control for both imidacloprid and fenitrothion assays (Table 1). Similarly, percentage of mortality 48 h post treatment was reportedly higher in F0 and F1 for both fenitrothion and imidacloprid, when compared with control bed bugs (Table 1). We found there is no significant difference between the susceptibility of control bed bugs and bed bugs treated with only B vitamins against all tested insecticides deltamethrin, fenitrothion, and imidacloprid, except an increase in the KT100 value for bed bugs treated with only B vitamins in imidacloprid assay (Table 1). Nonetheless, both control and B vitamins treated bed bugs were significantly more tolerant to imidacloprid when compared with antibiotic treated bed bugs (F0 and F1) (Table 1). These findings suggests that the addition of the B vitamins does not influence the effect of rifampicin treatment against the susceptibility of the bed bugs towards the insecticide.

**Table 1:** Restricted mean survival time (RMST) and knockdown response of *C. hemipterus* under 24 h continuous exposure and percentage mortality 48 h post-treatment against three different classes of insecticides.

Insecticide	Treatment Group	Time Point (min)	RMST (95% CI)	KT100 (min)	Percentage mortality 48 h post-treatment (%)
Deltamethrin (192 mg/m^2^)	Control	1440	1440.00 (1440.00–1440.00)^a^ SE = 0.00	> 1440.00^a^	0.00^a^
	F0		1440.00 (1440.00–1440.00)^a^ SE = 0.00	> 1440.00^a^	0.00^a^
	F1		1440.00 (1440.00–1440.00)^a^ SE = 0.00	> 1440.00^a^	0.00^a^
	Re-inoculated		1440.00 (1440.00–1440.00)^a^ SE = 0.00	> 1440.00^a^	0.00^a^
	B vitamin		1440.00 (1440.00–1440.00)^a^ SE = 0.00	> 1440.00^a^	0.00^a^
Imidacloprid (192 mg/m^2^)	Control	120	99.33 (91.83 -106.84)^ab^ SE = 3.83	186.67 ± 13.33^b^	66.67^ab^
	F0		73.33 (53.50—83.17)^c^ SE = 5.02	133.33 ± 6.67^d^	76.33^c^
	F1		63.33 (53.72—72.95)^c^ SE = 4.91	113.33 ± 6.67^d^	80.00^c^
	Re-inoculated		92.00 (82.84—101.17)^b^ SE = 4.68	160.00 ± 0.00^c^	73.33^bc^
	B vitamin		110.67 (104.07—117.26)^a^	220.00 ± 0.00^a^	63.33^a^
Fenitrothion (556 mg/m^2^)	Control	600	514.00 (479.97—548.03)^a^ SE = 17.36	940.00 ± 20.00^a^	16.67^a^
	F0		440.00 (404.79—475.21)^b^ SE = 17.96	620.00 ± 52.92^c^	43.33^b^
	F1		390.00 (354.18—425.82)^b^ SE = 18.28	560.00 ± 20.00^c^	56.67^b^
	Re-inoculated		506.00 (472.88—539.12)^a^ SE = 16.90	780.00 ± 34.61^b^	23.33^a^
	B vitamin		532.00 (497.95—566.05)^a^	1000 ± 20.00^a^	20.00^a^

Different letters in each row for each insecticide class indicate significant differences based on the comparison of restricted mean survival time (RMST) as determined by MedCalc software (*P* < 0.05) after Benjamini–Hochberg correction; Duncan’s multiple range test *P* < 0.05 for time to knockdown 100% bed bugs (KT100) and percentage mortality reported at 48 h post-treatment. Time point for RMST is pre-specified by Medcalc statistical software, set to the lowest time point of the last event among the different groups.

### Re-administration of microbial content from control individuals reduced susceptibility to insecticides in rifampicin-treated bed bugs

To further confirm contributions of microbiota mediating susceptibility of host bed bug to insecticides, we sought to determine whether tolerance to insecticides could be recapitulated by transfer of microbes from the whole-body crush of antibiotic-free individuals to the rifampicin treated bed bugs. Indeed, when the rifampicin treated bed bugs were fed with blood meal integrated with the whole-body crush of the control bed bug, RMST, KT100, and cumulative survival 48 h post exposure to fenitrothion and imidacloprid were significantly increased relative to F0 and F1 without the microbial transplant, except post 48 h mortality in imidacloprid assay (Table 1). The reinoculation of microbial content through the whole-body crushed blood meal also reverted the rifampicin treated bed bugs to be less susceptible to imidacloprid and fenitrothion like that observed in control bed bugs (Table 1).

### Metabolic enzyme activities of antibiotic-treated and untreated C. hemipterus

The enzymatic activities of α-esterase, β-esterase, total GST, and P450s from the whole-body lysates of bed bugs from the antibiotic-treated (F0 and F1) and control bed bugs were investigated to understand the relative association of xenobiotic metabolizing enzymes towards insecticide susceptibility in relation to the host’s microbiota. The results showed no significant differences in all enzyme assays between the control and rifampicin treated bed bugs (F0 and F1) (one-way ANOVA, α-esterase: df = 2, F = 0.712, *P* = 0.5; β-esterase: df = 2, F = 0.581, *P* = 0.566; total GST: df = 2, F = 0.076, *P* = 0.927; P450s: df = 2, F = 0.083, *P* = 0.921) (Supplementary Fig. S2).

### Analysis of 16S sequencing data and operational taxonomic units (OTUs)

A total of 2,945,945 raw reads were generated from all nine samples ranging from 212,797 to 454,703 reads, with a mean count of 327,327 reads per sample (Supplementary Table S1). Only reads with a quality score greater than 20 were selected, and the selected sequences were trimmed based on the length of primers (forward: 17 bp; reverse: 21 bp) and truncated to 270 bp and 250 bp respectively. A total of 273,061 high quality reads were obtained after quality trimming and filtering process, with the sequence count per sample ranged from 13,128 to 43,710 (mean 30,340). Subsequently, a total of 1354 reads affiliated to chloroplast, mitochondria, or without phylum assignation were removed from the filtered reads during taxonomic classification (Supplementary Table S1).

**Figure 2:**
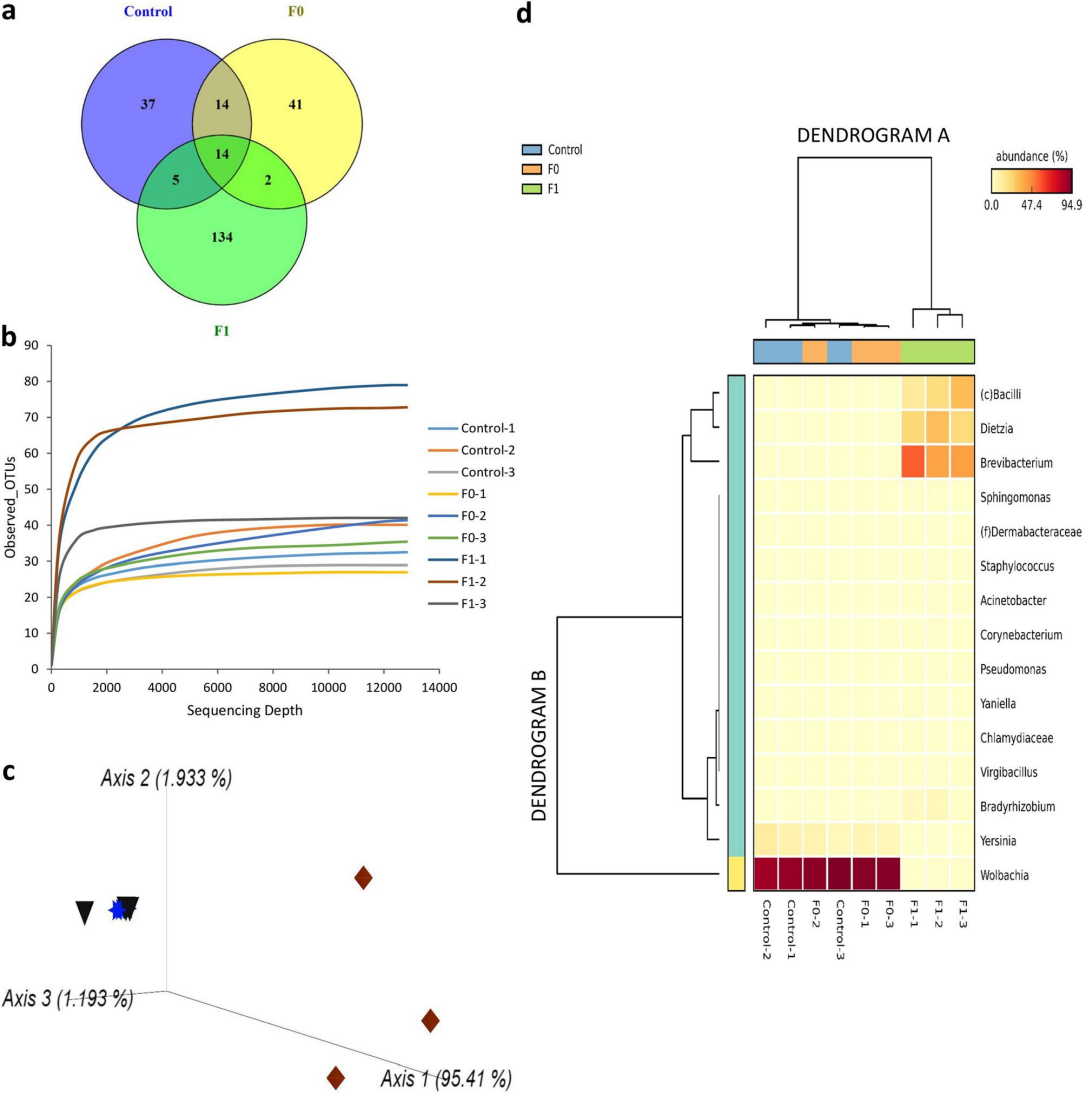
Effect of rifampicin treatment on microbial composition of *C. hemipterus* based on the 16S rRNA metagenomics analysis. (**a**) Venn diagram showing the number of shared and unique OTUs among the study groups; (**b**) Rarefaction curves of all samples from the study groups. Each line represents each replicate used in the respective study groups; (**c**) Principal coordinate analysis (PCoA) showing bacteria dissimilarity between the individuals from different experimental groups (rifampicin treated F0:blue star and F1: brown diamond ; control: black triangle) based on Bray–Curtis distance metric. Each symbol denotes individual replicate used in the respective study groups; (**d**) Heatmap dendrogram illustrating abundance (%) of top 15 genera in each sample from the three experimental groups (control, F0 and F1) and clustered by averaged neighbor UPGMA method at threshold of 0.95.

Overall, a total 247 OTUs (Supplementary Table S2) was detected from the three sample groups whereby 37 OTUs were unique to control bed bugs, 41 OTUs were unique to F0, and 134 OTUs were unique to F1, while the rest of the OTUs were shared among the three groups (Fig. 2a). Only 14 OTUs were shared among the three groups (Fig. 2a; Supplementary Table S2). F1 sample recorded the highest number of unique OTUs (134 OTUs).

### Microbial diversity in antibiotic-treated and untreated C. hemipterus

Rarefaction curves indicate that the number of reads generated for each of the sample was sufficient in describing the bacterial community composition (Fig. 2b). We observed no significant differences in Pielou’s evenness across the three sample groups (Kruskal Wallis test: H = 0.089, P = 0.96) (Table 2). Nonetheless, the Shannon’s diversity index (Kruskal Wallis H test, H = 7.2, *P* = 0.027), Fisher’s alpha (Kruskal Wallis H test, H = 5.42, *P* = 0.046), and Faith’s phylogenetic diversity (Kruskal Wallis H test, H = 6.49, *P* = 0.039) were found significant, in which highest diversity was observed in the F1 bed bugs, followed by F0 and control (Table 2). These findings suggest that although the relative proportions of the OTUs found within each group were similar, they may be dominated by different species groups.

**Table 2:** Effect of antibiotic treatment on alpha diversity of microbial communities in *C. hemipterus.*

Group	Fisher’s alpha	Shannon’s Index	Faith’s Phylogenetic Diversity	Pielou’s Evenness
Control	4.11 ± 0.44^a^	3.83 ± 0.01^a^	2.08 ± 0.14^a^	0.74 ± 0.02^a^
F0	3.96 ± 0.53^a^	3.79 ± 0.02^a^	2.14 ± 0.28^a^	0.75 ± 0.02^a^
F1	8.68 ± 1.67^b^	4.32 ± 0.07^b^	3.32 ± 0.41^b^	0.73 ± 0.03^a^

Different letters in each column indicate significant differences based on Kruskal–Wallis test followed by Dunn’s Test and Benjamini–Hochberg correction (*P* < 0.05).

Beta diversity analysis (Principal Coordinate Analysis, PCoA) based on the Bray–Curtis dissimilarity metric revealed clear clustering of microbial communities following rifampicin treatment (Fig. 2c). The findings showed a large proportion of the variability (> 95%) was accounted by the first three principal components. Two clusters were formed, in which control and F0 samples diverged greatly from the F1 samples indicating a significant shift in the microbial communities of the F1 bed bugs following a long-term course of rifampicin treatment (Fig. 2c).

### Effects of antibiotic treatment on the microbial community structure of C. hemipterus

#### Phylum and class level

We observed no significant differences in the microbial structure and community composition between the untreated control and F0 across all taxonomic levels (Table 3). On the contrary, divergences in the relative abundance of several microbial communities were significant (*P* < 0.05) between F1 and control across all taxonomic levels (Table 3). The microbiome of *C. hemipterus* from the three study groups can be classified into five major bacterial phyla, in which prevalence of sequences affiliated to the phylum Proteobacteria (> 99.0%) was observed in both control and F0 (Table 3). On the other hand, the phyla Actinobacteria (70.3%) and Firmicutes (22.4%) were significantly more abundant in F1 than control and F0 (*P* < 0.05) (Table 3). At the class level, Alphaproteobacteria (> 90.0%) were most abundant in the control and F0 followed by Gammaproteobacteria (6.45–8.01%) and were significantly more abundant than in F1. In contrast, the long-term rifampicin treatment significantly suppressed Alphaproteobacteria and Gammaproteobacteria in F1, and instead we observed an increase in the abundance of Actinobacteria, Bacilli, and Betaproteobacteria (Table 3).

**Table 3:** Mean relative abundance (%) ± SD of the predominant phyla, classes, orders, families, and genera found in *C. hemipterus* among the three study groups.

Taxa	Corrected *P*-value	Control	F0	F1
**Phylum (≥ 0.1%)**				
Proteobacteria	1.49E−09	99.20 ± 0.31^a^	99.20 ± 0.19^a^	6.93 ± 2.52^b^
Actinobacteria	1.76E−06	0.65 ± 0.41^b^	0.65 ± 0.39^b^	70.32 ± 6.26^a^
Firmicutes	0.0049	0.13 ± 0.09^b^	0.10 ± 0.13^b^	22.39 ± 8.26^a^
Chlamydiae	0.4219	0.00 ± 0.00	0.00 ± 0.00	0.23 ± 0.32
Bacteroidetes	0.5600	0.00 ± 0.00	0.05 ± 0.07	0.12 ± 0.17
**Class (≥ 0.1%)**				
Alphaproteobacteria	1.37E−08	91.19 ± 2.86^a^	92.75 ± 0.69^a^	4.53 ± 1.90^b^
Gammaproteobacteria	0.0218	8.01 ± 2.69^a^	6.45 ± 0.78^a^	1.78 ± 0.55^b^
Actinobacteria	1.42E−06	0.65 ± 0.41^b^	0.65 ± 0.39^b^	70.13 ± 6.02^a^
Bacilli	0.0056	0.13 ± 0.09^b^	0.08 ± 0.10^b^	22.25 ± 8.40^a^
Betaproteobacteria	1.29E−05	0.00 ± 0.00^b^	0.00 ± 0.01^b^	0.62 ± 0.08^a^
Chlamydiia	0.4219	0.00 ± 0.00	0.00 ± 0.00	0.23 ± 0.32
Coriobacteriia	0.4219	0.00 ± 0.00	0.00 ± 0.00	0.20 ± 0.28
Clostridia	0.4771	0.00 ± 0.00	0.02 ± 0.03	0.14 ± 0.20
Bacteroidia	0.4219	0.00 ± 0.00	0.00 ± 0.00	0.12 ± 0.17
**Order (≥ 0.2%)**				
Rickettsiales	3.15E−09	91.09 ± 2.75^a^	92.60 ± 0.72^a^	0.42 ± 0.31^b^
Enterobacteriales	0.0072	8.00 ± 2.69^a^	6.39 ± 0.77^a^	0.32 ± 0.14^b^
Actinomycetales	1.42E−06	0.65 ± 0.41^b^	0.65 ± 0.39^b^	70.13 ± 6.02^a^
Unnamed Bacilli	0.0049	0.11 ± 0.09^b^	0.00 ± 0.00^b^	21.23 ± 7.83^a^
Rhodobacterales	2.43E−06	0.01 ± 0.02^b^	0.00 ± 0.00^b^	0.30 ± 0.02^a^
Pseudomonadales	0.0003	0.01 ± 0.01^b^	0.03 ± 0.04^b^	1.11 ± 0.23^a^
Rhizobiales	0.0304	0.03 ± 0.03	0.12 ± 0.15	3.07 ± 1.64
Bacillales	0.0885	0.01 ± 0.01	0.00 ± 0.01	0.88 ± 0.64
Sphingomonadales	0.4219	0.03 ± 0.04	0.03 ± 0.04	0.56 ± 0.37
Burkholderiales	0.0790	0.00 ± 0.00	0.00 ± 0.00	0.45 ± 0.32
Chlamydiales	0.4219	0.00 ± 0.00	0.00 ± 0.00	0.23 ± 0.32
Coriobacteriales	0.4219	0.00 ± 0.00	0.00 ± 0.00	0.20 ± 0.28
**Family (≥ 0.2%)**				
Rickettsiaceae	3.07E−09	91.08 ± 2.74^a^	92.60 ± 0.71^a^	0.42 ± 0.31^b^
Enterobacteriaceae	0.0072	8.00 ± 2.69^a^	6.39 ± 0.77^a^	0.32 ± 0.14^b^
Brevibacteriaceae	5.85E−05	0.50 ± 0.29^b^	0.43 ± 0.25^b^	43.78 ± 7.10^a^
Dietziaceae	4.39E−05	0.13 ± 0.10^b^	0.17 ± 0.12^b^	24.54 ± 3.81^a^
Unnamed Bacilli	0.0049	0.11 ± 0.09^b^	0.00 ± 0.00^b^	21.23 ± 7.83^a^
Pseudomonadaceae	0.0108	0.00 ± 0.00^b^	0.00 ± 0.00^b^	0.45 ± 0.19^a^
Dermabacteraceae	0.0018	0.00 ± 0.00^b^	0.02 ± 002^b^	0.39 ± 0.11^a^
Bradyrhizobiaceae	0.0345	0.02 ± 0.02	0.04 ± 0.03	2.79 ± 1.56
Moraxellaceae	0.0552	0.01 ± 0.01	0.03 ± 0.04	0.66 ± 0.41
Staphylococcaceae	0.0781	0.01 ± 0.01	0.00 ± 0.00	0.44 ± 0.31
Bacillaceae	0.2023	0.00 ± 0.00	0.00 ± 0.01	0.44 ± 0.43
Yaniellaceae	0.1275	0.00 ± 0.00	0.02 ± 0.02	0.32 ± 0.26
Oxalobacteraceae	0.0849	0.00 ± 0.00	0.00 ± 0.00	0.24 ± 0.17
Corynebacteriaceae	0.4219	0.00 ± 0.00	0.00 ± 0.00	0.23 ± 0.32
Chlamydiaceae	0.4219	0.00 ± 0.00	0.00 ± 0.00	0.23 ± 0.32
Micrococcaceae	0.1263	0.01 ± 0.01	0.00 ± 0.00	0.23 ± 0.18
Coriobacteriaceae	0.4219	0.00 ± 0.00	0.00 ± 0.00	0.20 ± 0.28
**Genus (≥ 0.2%)**				
*Wolbachia*	3.13E−09	91.09 ± 2.75^a^	92.61 ± 0.71^a^	0.42 ± 0.31^b^
*Yersinia*	0.0058	7.99 ± 2.69^a^	6.36 ± 0.76^a^	0.00 ± 0.00^b^
*Brevibacterium*	5.78E−05	0.50 ± 0.29^b^	0.43 ± 0.25^b^	43.77 ± 7.08^a^
*Dietzia*	4.57E−05	0.13 ± 0.10^b^	0.17 ± 0.12^b^	24.51 ± 3.83^a^
Unnamed *Bacilli*	0.0049	0.11 ± 0.09^b^	0.00 ± 0.00^b^	21.23 ± 7.83^a^
*Acinetobacter*	0.0023	0.01 ± 0.01^b^	0.03 ± 0.04^b^	0.46 ± 0.14^a^
Unnamed *Dermabacteraceae*	9.15E−05	0.00 ± 0.00^b^	0.02 ± 002^b^	0.28 ± 0.04^a^
*Bradyrhizobium*	0.0345	0.02 ± 0.02	0.04 ± 0.03	2.79 ± 1.56
*Virgibacillus*	0.2024	0.00 ± 0.00	0.00 ± 0.01	0.44 ± 0.43
*Pseudomonas*	0.0888	0.00 ± 0.00	0.00 ± 0.00	0.47 ± 0.20
*Staphylococcus*	0.0638	0.01 ± 0.01	0.00 ± 0.00	0.36 ± 0.24
*Sphingomonas*	0.0718	0.03 ± 0.04	0.03 ± 0.04	0.36 ± 0.22
*Yaniella*	0.1275	0.00 ± 0.00	0.02 ± 0.02	0.32 ± 0.26
Unnamed *Caulobacteraceae*	0.4623	0.04 ± 0.05	0.00 ± 0.00	0.29 ± 0.42
*Corynebacterium*	0.4219	0.00 ± 0.00	0.00 ± 0.00	0.23 ± 0.32
*Chlamydiaceae*	0.4219	0.00 ± 0.00	0.00 ± 0.00	0.23 ± 0.32
*Enhydrobacter*	0.4219	0.00 ± 0.00	0.00 ± 0.00	0.20 ± 0.28

Different letters in the same row indicate statistical significance between taxonomic abundance based on STAMP analysis using ANOVA followed by Tukey–Kramer and Benjamini–Hochberg correction (*P* < 0.05).

#### Order and family level

At the order level, divergences in the relative abundance of the OTUs was found in six orders including Rickettsiales, Enterobacteriales, Actinomycetales, Unnamed Bacilli, Rhodobacterales, and Pseudomonodales (Table 3). Rickettsiales was most dominant (> 90%) followed by Enterobacteriales in control and F0 (Table3). In contrast, their suppression resulted in a significant increase of Actinomycetales, Unnamed Bacilli, Pseudomonadales, and Rhodobacterales in the F1, in which Actinomycetales (70.1%) and Unnamed Bacilli (21.2%) were most abundant (Table 3). Similarly, Rickettsiaceae and Enterobacteriaceae were the most dominant family in the control and F0 groups which made up to approximately 99% of their bacterial community (Table 3). On the other hand, Brevibacteriaceae (43.8%), Dietziaceae (24.5%), and Unnamed Bacilli (21.2%) were significantly increased in F1 (Table 3). Although Pseudomonadacea and Dermabactereraceae only represent a small portion among the bacterial families in F1, there was a significant increase in their abundance relative to both control and F0 (Table 3).

#### Genus level

To provide an overview on the bacterial community structure at the genus level between the samples, a heatmap analysis of 15 most abundant bacterial genera was generated based on the UPGMA clustering at 0.95 threshold level (Fig. 2d). Dendrogram A (Fig. 2d) indicated two major clusters; one cluster comprising all F1 samples, while the other comprising remaining six samples from the control and F0, suggesting a clear distinction in the bacterial genera community profile between F1 and control as well as with F0. On the other hand, dendrogram B segregated the bacterial composition into two major clusters based on their relative abundance (Fig. 2d). The first cluster was unique and represented by the genus *Wolbachia* (91.1–92.6%) that was significantly higher in both control and F0 than the F1 bed bugs (*P* < 0.05) (Table 3). The second cluster was divided into two subclusters; a *Yersinia* (6.4–8.0%) dominant subcluster that was highly represented in control and F0 and another subcluster dominated by the genera *Brevibacterium* (43.8%), *Dietzia* (24.5%), and an unknown genus of Bacilli (21.3%) that were found significantly higher in F1 than control and F0 (*P* < 0.05) (Table 3).

### Functional prediction analysis

**Figure 3:**
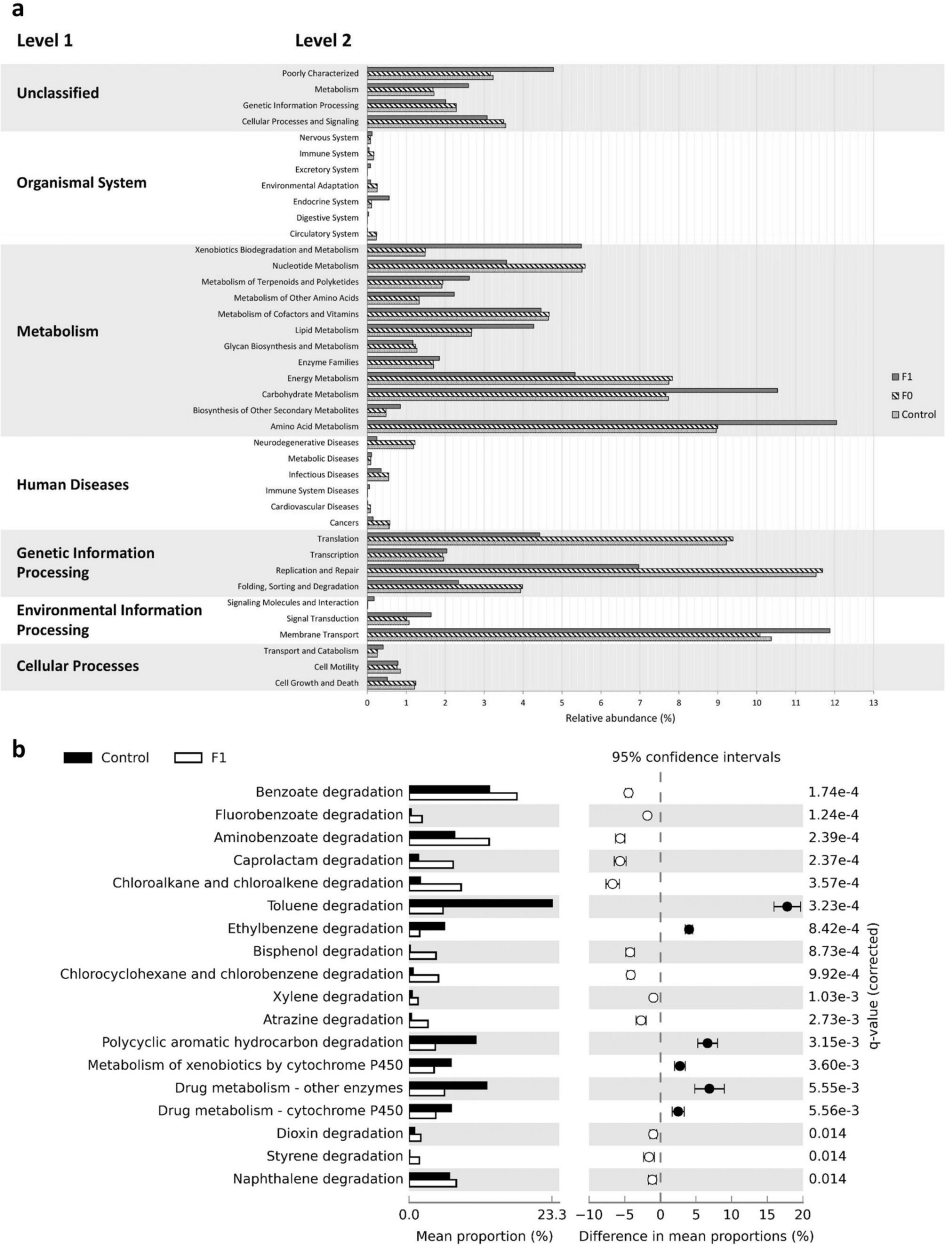
Functional prediction analysis generated by PICRUSt. (**a**) The relative abundance of the predicted functions grouped according to KEGG level 1 and 2 categories of the bacterial communities; (**b**) Extended error bar of predicted function subsystem of xenobiotics biodegradation and metabolism at KEGG level 3. The results were filtered using a Welch’s t-test (two groups comparison) with Benjamini–Hochberg FDR correction (*P*-value < 0.05) in STAMP v2.1.3.

Subsequently, a metagenome prediction approach was used to determine probable functions of the bacterial communities in *C. hemipterus* through the PICRUSt software package, which implements 16S rRNA libraries to make functional prediction of the metagenome^41^. The relatively small nearest sequenced taxon index (NSTI) values ranging from 0.0213 to 0.0302 for the nine samples indicated the accuracy of PICRUSt metagenome predictions in our study^41^. The predicted protein-coding genes were categorized by function using the KEGG GO level 1 to 3. The KEGG level 1 pathways indicated a high abundance of predicted functions related to metabolism (45.6–54.5%), genetic information processing (15.8–27.0%), and environmental information processing (11.1 – 13.7%) in all three groups (Fig. 3a). However, the predicted functions of F1 were significantly different in relative frequency when compared with control and F0 for all KEGG level 1 functional categories (Table 4); environmental information processing, metabolism, and organismal system were significantly more abundant in F1 whereas functions related to cellular processes, genetic information processing, and human diseases were significantly higher in the control and F0 groups. The KEGG level 2 data (Fig. 3a; Table 4) further revealed that 36 of the total 39 functional categories were differentially predicted across the three sample groups, of which F1 predicted functions were significantly different than F0 and control in all 36 categories (Fig. 3a; Table 4). More specifically, 20 pathways were significantly enriched in both control and F0, whereas 16 were enriched in F1 (Table 4; Fig. 3a). Despite the differences, membrane transport, replication and repair, amino acid metabolism, carbohydrate metabolism, and energy metabolism remain most prevalent (> 5% within each category) functional categories across the three samples (Table 4; Fig. 3a). Within the metabolism category (i.e., the most abundant category), amino acid metabolism, biosynthesis of other secondary metabolites, carbohydrate metabolism, enzyme families, lipid metabolism, metabolism of other amino acids, metabolism of terpenoids and polyketides, and xenobiotics biodegradation and metabolism were enriched in F1, whilst energy metabolism, metabolism of cofactors and vitamins, and nucleotide metabolism were enriched in both control and F0 (Table 4; Fig. 3a). More specifically, we have targeted the pathways belonging to xenobiotics biodegradation and metabolism in KEGG level 3 to identify potential pathways related to insecticide tolerance differences between the antibiotic treated and untreated bed bugs (Fig. 3b). Based on STAMP analysis, the untreated control showed significantly higher relative abundance in toluene degradation, ethylbenzene degradation, polycyclic aromatic hydrocarbon degradation, metabolism of xenobiotics by cytochrome P450, and drug metabolism (cytochrome P450 and other enzymes) functions when compared to rifampicin treated F1, which could be involved in the degradation of insecticides in the control bed bug resulting in higher tolerance to fenitrothion and imidacloprid (Fig. 3b).

**Table 4:** Mean relative abundance of the predicted functions of the bacterial communities grouped according to KEGG pathway categories among the three study groups.

Level 1	Level 2	Corrected *P*-value	Control	F0	F1
Cellular Processes	Cell Growth and Death	6.54E−07	1.21 ± 0.03^a^	1.25 ± 0.02^a^	0.51 ± 0.04^b^
	Cell Motility	0.8015	0.85 ± 0.09	0.76 ± 0.05	0.79 ± 0.22
	Transport and Catabolism	3.43E−06	0.26 ± 0.00^b^	0.26 ± 0.00^b^	0.41 ± 0.01^a^
Environmental Information Processing	Membrane Transport	0.0006	10.37 ± 0.30^b^	10.08 ± 0.16^b^	11.88 ± 0.24^a^
	Signal Transduction	2.94E−07	1.08 ± 0.07^b^	1.01 ± 0.03^b^	1.64 ± 0.02^a^
	Signaling Molecules and Interaction	3.92E−06	0.01 ± 0.00^b^	0.01 ± 0.00^b^	0.17 ± 0.00^a^
Genetic Information Processing	Folding, Sorting and Degradation	1.65E−07	3.94 ± 0.06^a^	3.98 ± 0.02^a^	2.34 ± 0.08^b^
	Replication and Repair	8.44E−06	11.52 ± 0.19^a^	11.69 ± 0.09^a^	6.97 ± 0.30^b^
	Transcription	0.0097	1.96 ± 0.02	1.94 ± 0.01	2.04 ± 0.05
	Translation	1.97E−08	9.22 ± 0.19^a^	9.39 ± 0.09^a^	4.43 ± 0.22^**b**^
Human Diseases	Cancers	1.02E−07	0.56 ± 0.02^a^	0.58 ± 0.01^a^	0.15 ± 0.02^b^
	Cardiovascular Diseases	3.35E−06	0.08 ± 0.00^a^	0.08 ± 0.00^a^	0.01 ± 0.01^b^
	Immune System Diseases	5.65E−07	0.01 ± 0.00^b^	0.01 ± 0.00	0.06 ± 0.00^a^
	Infectious Diseases	2.15E−09	0.55 ± 0.00^a^	0.55 ± 0.00^a^	0.36 ± 0.01^**b**^
	Metabolic Diseases	1.00E−07	0.09 ± 0.00^b^	0.09 ± 0.00^b^	0.11 ± 0.11^a^
	Neurodegenerative Diseases	0.0006	1.19 ± 0.04^a^	1.22 ± 0.02^a^	0.25 ± 0.03^b^
Metabolism	Amino Acid Metabolism	5.69E−10	8.96 ± 0.04^b^	9.00 ± 0.03^b^	12.05 ± 0.06^a^
	Biosynthesis of Other Secondary Metabolites	5.28E−07	0.48 ± 0.01^b^	0.48 ± 0.00^b^	0.85 ± 0.03^a^
	Carbohydrate Metabolism	1.09E−07	7.73 ± 0.08^b^	7.66 ± 0.04^b^	10.53 ± 0.14^a^
	Energy Metabolism	7.83E−08	7.74 ± 0.10^a^	7.83 ± 0.05^a^	5.33 ± 0.07^b^
	Enzyme Families	0.0021	1.70 ± 0.01^b^	1.69 ± 0.01^b^	1.85 ± 0.05^a^
	Glycan Biosynthesis and Metabolism	0.0097	1.28 ± 0.03^a^	1.24 ± 0.02^a^	1.17 ± 0.01^b^
	Lipid Metabolism	1.00E−07	2.67 ± 0.01^b^	2.68 ± 0.00^b^	4.27 ± 0.09^a^
	Metabolism of cofactors and vitamins	0.0359	4.65 ± 0.02^a^	4.67 ± 0.01^a^	4.46 ± 0.11^b^
	Metabolism of other amino acids	6.75E−07	1.33 ± 0.01^b^	1.33 ± 0.00^b^	2.33 ± 0.07^a^
	Metabolism of terpenoids and polyketides	5.54E−07	1.91 ± 0.02^b^	1.94 ± 0.01^b^	2.62 ± 0.05^a^
	Nucleotide metabolism	1.04E−06	5.51 ± 0.09^a^	5.59 ± 0.04^a^	3.57 ± 0.13^b^
	Xenobiotics biodegradation and metabolism	1.42E−06	1.49 ± 0.03^b^	1.49 ± 0.01^b^	5.49 ± 0.38^a^
Organismal Systems	Circulatory system	1.75E−08	0.23 ± 0.01^a^	0.24 ± 0.00^a^	0.01 ± 0.00^b^
	Digestive system	6.41E−08	0.00 ± 0.00^b^	0.00 ± 0.00^b^	0.03 ± 0.00^a^
	Endocrine system	2.42E−07	0.11 ± 0.01^b^	0.11 ± 0.00^b^	0.56 ± 0.03^a^
	Environmental adaptation	4.17E−09	0.25 ± 0.00^a^	0.26 ± 0.00^a^	0.09 ± 0.00^b^
	Excretory system	2.65E−10	0.00 ± 0.00^b^	0.00 ± 0.00^b^	0.08 ± 0.00^a^
	Immune system	1.65E−07	0.16 ± 0.00^a^	0.17 ± 0.00^a^	0.05 ± 0.00^b^
	Nervous system	8.44E−06	0.08 ± 0.00^b^	0.08 ± 0.00^b^	0.13 ± 0.00^a^
Unclassified	Cellular processes and signaling	0.0002	3.65 ± 0.05^a^	3.50 ± 0.03^a^	3.08 ± 0.07^b^
	Genetic information processing	8.44E−06	2.28 ± 0.00	2.28 ± 0.00	2.01 ± 0.03
	Metabolism	3.92E−06	1.71 ± 0.03^b^	1.69 ± 0.01^b^	2.60 ± 0.09^a^
	Poorly characterized	0.0359	0.00 ± 0.00^b^	3.16 ± 0.04^b^	4.78 ± 0.11^a^

Different letters in the same row indicate statistical significance between taxonomic abundance of predicted functions based on STAMP analysis using ANOVA followed by Tukey–Kramer and Benjamini–Hochberg correction (*P* < 0.05).

## Discussion

The association between bacterial symbionts and insecticide susceptibility in bed bugs has not been described. It is possible insecticides selection pressure may result in resistant insects harboring microbes that contribute to increased survival under this pressure. In this study, we demonstrated that the microbiota of *C. hemipterus* influence its susceptibility to imidacloprid; a neonicotinoid insecticide that act on the central nervous system of insects by blocking nicotinic acetylcholine receptors, and fenitrothion; a synthetic organophosphate acetylcholinesterase inhibitor and endocrine disrupter that is used as an insecticide. The disruption of the microbiota through rifampicin antibiotic treatment significantly reduced survival of the bed bugs when subjected to imidacloprid and fenitrothion in both F0 and F1 bed bugs. This finding suggests that a single application of rifampicin is sufficient to demonstrate the effect of microbiota disruption on the susceptibility of bed bugs against the insecticides. The rifampicin treatment, however, did not affect the reproductive and developmental fitness of the bed bugs when provided with blood meals containing B vitamins, which suggests the increased susceptibility against imidacloprid and fenitrothion were likely influenced by the bacterial composition and their metabolic dynamics during dysbiosis caused by the antibiotic treatment.

Conversely, the survival of the rifampicin treated bed bugs was not significantly affected when subjected to deltamethrin; a pyrethroid ester insecticide that acts upon the voltage-gated sodium channels (VGCS) in the axonal membranes of insects. The *C. hemipterus* strain used in the current study is known to carry the M918I and L1014F mutations in the VGSC genes^42^ that have been previously associated with high levels of knockdown resistance (*kdr*) against pyrethroids in *C. hemipterus*^26,37^ and other insect species^43,44^*.* It is probable that the resistance from VGSC mutations against pyrethroid insecticide cannot be suppressed by the disruption of the microbiota through antibiotics treatment. Furthermore, the 16S rRNA metagenomics sequencing of the *C. hemipterus* microbiome provided empirical evidence on bacterial communities potentially involved in mediating the susceptibility of *C. hemipterus* against imidacloprid and fenitrothion. As it stands, the rifampicin treatment significantly alters the bacterial assemblage in F1 bed bugs, resulting in enhanced susceptibility against imidacloprid and fenitrothion.

The use of chemical pesticides has been long associated with the development of resistance in insects, and reports on the role of bacterial symbionts in mediating insecticide susceptibility has exploded only after the first observation of Boush and Matsumura^45^ demonstrating the role of *Pseudomonas melophthora* in degrading multiple pesticide class including organophosphate, organochlorine, and carbamate was revisited 45 years later by Kikuchi et al.^39^. The findings of Kikuchi et al.^39^ confirmed the functional role of symbiont mediating insecticide susceptibility and showed that this phenomenon can also be found in other bacterial taxa such as *Burkholderia* sp. that degrades fenitrothion and diazinon. Since then, many observations have been observed across various insect groups involving different bacterial taxa such as in the association between *Bactrocera dorsalis* and *Citrobacter* sp. against the trichlorphon insecticide^46^, *Plutella xylostella* and *Enterococcus* sp. against the chlorpyrifos insecticide^47^, and *Nilaparvata lugens* and *Arsenphonus* sp. against the imidacloprid and buprofezin insecticides^48^. The advancement of culture-independent technologies such as 16S metagenomics sequencing had further facilitated the discovery of gut microbiota associated with insecticide susceptibility of the host insect, which suggested the association may not necessarily be specific to a single bacterial taxon but the microbiota community within the host^49,50^. Akami et al.^49^ demonstrated that the presence of dominant gut bacterial phyla Proteobacteria, Bacteroidetes, and Firmicutes especially from the genera *Acinetobacter, Citrobacter*, *Pseudomonas* and Burkholderiales (unclassified genera) potentially involved in the increased tolerance of the host cowpea beetle, *Callosobruchus maculatus* against the dichlorvos insecticide (DDVP) based on the 454-pyrosequencing approach. Similarly, Pietri et al.^50^ reported that antibiotic treatment (doxycycline) shifted the microbiota composition of *Blatella germanica* bait-selected resistant strain towards one that more closely resembled that of the susceptible strain such as Clostridia (Firmicutes), Deltaproteobacteria (Proteobacteria), and Bacteroidia (Bacteroidetes), resulting in higher mortality of the treated insects to indoxacarb. In our samples, we also observed a drastic reduction in the abundance Proteobacteria, particularly Alphaproteobacteria and Gammaproteobacteria in rifampicin treated F1 bed bugs which resulted in increased susceptibility to fenitrothion and imidacloprid. Instead, we found a higher abundance of Actinobacteria and Firmicutes (Bacilli) in F1 bed bugs which were previously found in low abundance in untreated control (Table 3).

More specifically, we found that the disruption of the abundant *Wolbachia* and possibly *Yersinia* could be related to the enhanced susceptibility towards the insecticides. Nonetheless, it is noteworthy that *Wolbachia* is an obligate endosymbiont in the common bed bug, *C. lectularius*, essential for B vitamins provisioning for development and growth^3,15^, therefore, all individuals in a bed bug population would be expected to be infected by *Wolbachia*. However, the exact role of *Wolbachia* in the tropical bed bug, *C. hemipterus*, is yet to be described. Currently, there is limited evidence on the role of *Wolbachia* in mediating insecticide susceptibility in insects. The association of *Wolbachia* infection and insecticide susceptibility in insects can be double-edged. On the one hand, some studies proposed that *Wolbachia* infection can be associated with increased tolerance to insecticide such as recently observed in the hemipteran insect, *Laodelphax striatellus*^51^. Eliminating the *Wolbachia* from the buprofezin-resistant strain would enhance susceptibility of the *L. striatellus* to buprofezin^51^, which corroborates the findings from our present study. However, the association of *Wolbachia* with insecticide susceptibility was restricted to some genetic backgrounds of the host insects while having no effect in other backgrounds^52^. On the other hand, a high density of *Wolbachia* was found to exert fitness cost on the host insects resulting in decreased tolerance to insecticide such as observed in the whitefly, *Bemicia tabaci*^53^ and the mosquito, *Culex quinquefasciatus*^54^. Conversely, Berticat et al.^55^ reported that *Culex pipiens* mosquitoes may control *Wolbachia* density less efficiently (i.e., having higher infection rate) when they carry insecticide-resistant genes, suggesting effects of the host genome on the endosymbiont levels. Therefore, a clear-cut distinction whether host genetics (i.e., when they suffer from physiological cost by carrying resistance genes) influences *Wolbachia* infection or *Wolbachia* infection influences susceptibility of host insects against insecticides are yet to be ruled out.

Alternatively, the symbiont-mediated insecticide susceptibility in bed bugs may not be caused by the presence of a singular taxa or species, but rather depends on the diversity and complex interactions of the microbiota and their metabolic dynamics that can be disrupted/altered by the antibiotic treatment. In the present study, the use of rifampicin may have selectively removed certain groups of bacteria susceptible to its activity. Testing for symbionts mediating insecticide susceptibility using antibiotics with different range of activity would help corroborating the findings as well as identify the bacteria responsible. We noticed that rifampicin treatment did not significantly alter the bacterial composition of F0 bed bugs when compared to the untreated bed bugs. Rifampicin belongs to the rifamycin group of antibiotics. It works by decreasing the production of RNA by bacteria by inhibiting bacterial DNA-dependent RNA polymerase, thus preventing synthesis of host bacterial proteins^56,57^. Apparently, a single application of rifampicin may have arrested the metabolic activities of the afflicted bacteria resulting in increased susceptibility of the host bed bugs towards imidacloprid and fenitrothion. Nonetheless, traces of genomic DNA of the afflicted bacteria remains viable (i.e., the bacteria were eventually dead, however, the genomic material has not been cleared out) and can be detected in the 16S metagenomics sequencing of the single feeding, F0 bed bugs. The effect of rifampicin on the genomic integrity of the afflicted bacteria only becomes more apparent in the F1 bed bugs that received a continuous course of the antibiotic.

We also observed that continuous rifampicin treatment that largely removes *Wolbachia* increases the bacterial diversity in F1 bed bugs. This finding is consistent with the report by Duan et al.^58^ that shows the proportions of *Wolbachia* infections significantly correlates with bacterial diversity in small brown plant hopper (SPBH), whereby Wolbachia infection severely decreases the diversity and abundance of bacteria in SBPH. Similarly, this phenomenon was also observed in *Aedes aegypti*, in which a large proportion of bacterial taxa disappeared when *Wolbachia* was induced by artificial injection^59^. Also, a very low bacterial diversity was found in the gut of *Drosophila melanogaster*, which is naturally infected with *Wolbachia*^60^. Apart from this, there is a possibility for the rifampicin treated F1 bed bugs to also acquire bacterial taxa from the environment during the experimental period. The F0 bed bugs have shorter exposure time following rifampicin treatment (7 days) before being subjected to analysis, however the F1 bed bugs experienced an extended experimental period allowing those suppressed rare/transient taxa within the bug and potentially, newly acquired taxa from the environment (i.e., previously unable to establish colonies within bed bugs due to the presence of dominant Wolbachia) to flourish, therefore resulting in increased diversity.

The shift in the microbial assemblage between the untreated control and rifampicin treated bed bugs also reflected significant changes in the predicted functions of the bacteria. Unexpectedly, we observed higher proportion of functions related to xenobiotics biodegradation and metabolism category enriched in the F1 relative to the untreated bed bugs (Fig. 3a). This observation could be accounted by the significant increase in the diversity of the bacteria with varied functions in F1 following rifampicin treatment. Due to the limitations of the functional prediction analysis based on short reads of 16S rRNA, we are unable to conclusively say if there is a correlation between increased diversity of predicted bacterial metabolic function correspond to the reduction in insecticide tolerance. However, the increased diversity of bacterial metabolic function in antibiotic treated bed bugs is consistent with the findings of Duan et al.^58^ that showed presence of *Wolbachia* affected the overall expression of metabolism genes of SBPH to suppress the diversity/abundance of bacterial populations. Removal of *Wolbachia* therefore increases the bacterial diversity, which in turn increases the diversity of bacterial metabolic functions as observed in the present study. Nonetheless, we observed functions related to toluene degradation, ethylbenzene degradation, polycyclic aromatic hydrocarbon degradation, metabolism of xenobiotics and drug metabolism by cytochrome P450 and other enzymes were enriched in untreated control and significantly reduced function in the F1 bed bugs (Fig. 3b), which likely contribute to the increased susceptibility to imidacloprid and fenitrothion in F1 bed bugs. Both imidacloprid and fenitrothion insecticides contain derivatives of aromatic hydrocarbon rings, and cytochrome P450 has been previously described to confer metabolic resistance against organophosphate in bed bugs^34^ and neonicotinoid in other insects such as in the aphid^61^ through synergism studies.

Furthermore, Tang et al.^62^ reported that bacterial symbionts including *Wolbachia*, *Arsenophonus, Acinetobacter*, and *Staphylococcus* may affect the effectiveness of the insecticide by regulating the expression of the insect host’s GST and P450 genes. However, we observed no significant changes in the enzymatic activities of total P450, esterase, and GST extracted from the whole-body lysate of *C. hemipterus* across all test conditions. This is likely because the whole-body lysate approach measures a change in total enzymatic activity and may not be sensitive to detect specific changes in the microbial enzymes during dysbiosis that could contribute to the insecticide detoxification. Alternatively, the microbial detoxification of the insecticide may involve other candidate proteins/enzymes such as glucosidase, phosphatase, hydrolase, dioxygenases, peroxidases, and laccases^40,63^, however, cannot be distinguished in the present study due to the limitations of the functional predictions based on16S rRNA data. Therefore, methods that compares microbial RNA from insecticide susceptible and resistant bed bugs should be explored further which may help identify microbial genes responsible for mediating insecticide susceptibility in insects, especially when concerning non-culturable bacteria^38^.

Lastly, the microbiota transplant experiment via crushed whole-body of antibiotic-free bed bugs demonstrated that the microbial community along with the associated traits can be passed to the rifampicin treated individuals to regain tolerance against the imidacloprid and fenitrothion in a single feeding, further corroborating the role of microbes in mediating insecticide susceptibility in bed bugs. The current transplanting approach (i.e., culture independent, crushed whole body) may not be able to distinguish if the observed changes were primarily due to the bacterial symbiont and/or caused by other non-bacterial symbionts, which requires further investigations. Nonetheless, similar observations of microbial transplant experiments enhancing tolerance to insecticides to otherwise susceptible insects were reported in other insects such as the German cockroach^50^ through fecal transplant, and the findings have been associated with the gut bacterial community. On the other hand, direct inoculation of isolated bacteria resulting in enhance tolerance to insecticides has also been reported elsewhere such as in the plant hopper through oral feeding of bacteria suspended leaves^48^ and silkworm through oral inoculation of bacteria^64^.

In conclusion, the present study has demonstrated that the bacterial symbionts of *C. hemipterus* significantly influence its susceptibility to insecticides. The mechanisms involved in this correlation is currently unknown and likely dependent on the complex interactions of the symbionts with the host’s genetics background and multiple resistance mechanisms including target site insensitivity, penetration resistance, and metabolic resistance that should be the focus of future studies. The relevance of our findings in the management of insecticide resistant bed bugs in the field remains to be tested. Currently, the chemical control option in bed bugs heavily rely on the use of contact insecticides and residual formulations including pyrethroid, neonicotinoid, and organophosphate which may not work effectively with antibacterial compounds. On the other hand, most available bed bugs baiting products rely on sticky traps and odor and/or heat perception that offers little advantage in incorporating antimicrobial compounds. Nonetheless, this study provides new knowledge for devising alternative strategies in managing the insecticide resistant bed bugs by targeting the microbiota of the resistant bed bugs. Lastly, the 16S rRNA metagenomics data from the present study also provided useful resources on the microbial assemblage of the *C. hemipterus* bacterial endosymbionts which is currently lacking.

## Methods

### Bed bugs rearing and maintenance

A previously described pyrethroid resistant population of *C. hemipterus*, SEL_MY collected from a dormitory in Puchong, Selangor, Malaysia^42^ was used throughout the experiments. The bed bug cultures were maintained in the laboratory under standard conditions of 27 ± 2 °C and 70 ± 5% relative humidity with a photoperiod of 12:12 (L:D) h. The bed bugs were fed on fresh defibrinated rabbit blood through a parafilm-membrane feeding system which was maintained at 37 °C on a water bath once a week.The rabbit blood was provided by the Animal Research and Service Centre, (ARASC), Universiti Sains Malaysia withdrawn from the artery of rabbit’s ears and stored in lithium heparin tubes prior feeding. The protocol for blood collection from the rabbit for blood-feeding activity was reviewed and approved by the Universiti Sains Malaysia Animal Ethics Committee [USM/Animal Ethics Approval/2016/(104)(819)].

### Antibiotic treatment

To understand the influence of bacterial symbionts on insecticide susceptibility, the study bed bugs were treated with an antibiotic agent (rifampicin) (Gold Biotechnology, USA, Cat. No. R-120-5) as described by Hosokawa et al*.*^3^ The rifampicin was suspended in methanol (solvent) and added into the blood meal to a final concentration of 10 μg/mL. Since rifampicin treatment would remove *Wolbachia*; an obligate nutritional mutualist that provides B vitamins essential for the growth and development of bed bugs^3^, we supplemented the rifampicin-incorporated defibrinated rabbit blood meal with a B vitamin complex (5 μL /mL; Nature’s Answer, USA, Product ID: 26107) to supply essential nutrients for the bed bugs^3^. Three experimental setups were implemented: (i) Control—fifth instar nymphs fed once with methanol-treated blood meal; (ii) F0—fifth instar nymphs fed once with rifampicin-treated blood meal supplemented with B vitamins; (iii) F1—the F1 generation from the F0 parents were fed continuously with rifampicin-treated blood meals supplemented with B vitamins until adult emergence. Additionally, we also investigated the effect of B vitamins on insecticide susceptibility by subjecting the bed bugs treated with only B vitamins in their blood meal without the antibiotics (i.e., fifth instar nymphs fed once with B vitamins-treated blood meal) to insecticide bioassay against the study insecticides. This is to ensure the observed changes in the susceptibility is due to the antibiotic treatment and not because of the B vitamins supplement. In all cases, the bed bugs were fed to repletion and monitored daily for adult emergence. Only male, unmated, seven-day-old adult bed bugs were used for the subsequent insecticide bioassay, metabolic enzyme biochemical assay, and DNA extraction for the 16S rRNA amplicon sequencing as described below.

### Fitness effect of rifampicin treatment against C. hemipterus

The relative impact of rifampicin treatment on the fitness of the bed bugs was evaluated based on the oviposition activity, egg hatchability, and nymphal development from egg to adult. Virgin male and female bed bugs were obtained by isolating recently fed fifth instar nymphs from the stock culture until emergence into adults. In the treatment group, the unmated male and female bed bugs were paired and allowed to feed once a week on blood meals supplemented with 10 μg/mL rifampicin and B vitamins as described above, for up to eight weeks. The number of eggs deposited, and its hatchability was observed each week for each pair of the bed bugs and the results were averaged. Subsequently, the emerging first instar nymphs were collected and reared on rifampicin and B vitamins supplemented blood meals on a weekly basis until they reached the adult stage. The time taken for the nymphs to molt to the next instar stage was monitored and recorded until all nymphs emerged into adults. The exuviae of each nymph was used to indicate that molting has taken place. Individuals that did not feed were excluded from the experiment. For the control, same data were collected from bed bugs that were fed on blood meals with methanol only. Ten pairs of bed bugs were used for both treatment and control groups.

### Insecticide bioassay

Three analytical grade insecticides, namely deltamethrin (≥ 98.0%, PESTANAL, Sigma-Aldrich, Munich, Germany), imidacloprid (≥ 98.0%, PESTANAL, Sigma-Aldrich, Munich, Germany), and fenitrothion (≥ 95.0%, PESTANAL, Sigma-Aldrich, Munich, Germany) were used in this study. The insecticide bioassay was adopted from the glass Petri dish assay method previously described by Dang et al.^65^. Acetone-diluted insecticide (0.5 mL) was applied evenly onto a glass Petri dish (90 mm diameter × 15 mm height) based on the discriminating concentration of respective insecticides (192 mg AI m^-2^ for both deltamethrin and imidacloprid^65^ and 556 mg AI m^-2^ for fenitrothion^31^). The insecticide-treated Petri dishes were left overnight to dry in a fume hood. Control Petri dishes were treated with acetone only. Three replicates, each with ten male bed bugs, were subjected to each of the insecticides and control treatments. The cumulative number of knocked down bed bugs were recorded at regular time intervals for up to 24 h. The bed bug was considered knocked down if it unable to move or right itself within 15 s even after being gently touched with a fine forceps. Knocked down individuals were transferred to a clean Petri dish and mortality was assessed at 48 h post-treatment.

### Re-inoculation of microbial content from crushed whole body of untreated bed bugs to rifampicin-treated C. hemipterus colony

Cohorts of the fifth instar nymphs were isolated from the colony and fed to repletion with blood meal supplemented with rifampicin and B vitamins. The fed fifth instar nymphs were allowed to molt into the adult stage and sexed. The male bed bugs were then subjected to a blood meal containing crushed whole body of a surface sterilized adult male bed bug from an untreated (antibiotic-free) colony. The male bed bugs were then aged for seven days without any further blood meals prior to subjecting them to insecticide bioassays as described above.

### Metabolic enzyme biochemical assays

Bed bugs were homogenized individually in 600 µL of ice-cold 0.1 M phosphate buffer (pH 7.0) containing 0.3% Triton X-100 (v:v) and subsequently centrifuged at 10,000 *g* for 10 min at 4 °C. The supernatant was collected and transferred to a new tube. Ten biological replicates (one individual per replicate), each with five technical replicates were used for rifampicin-treated and untreated bed bugs in each biochemical assay. Blank samples without the homogenate were included in each run to serve as the negative control. Total protein content was determined based on the Bradford method^66^. Determination of general esterase activity and GST assay was done according to procedures described in Khalid et al.^67^, whereas P450s heme peroxidase activity was determined according to Son-un et al.^68^ with modifications. A detailed description of the methods used is provided in the Supplementary Methods.

### DNA extraction and 16S rRNA amplicon sequencing

Metagenomic DNA was extracted from the whole insect body using FavorPrep Tissue Genomic DNA Extraction Mini Kit (FAVORGEN Biotech Corporation, Ping-Tung, Taiwan) following the manufacturer’s protocol. Prior to DNA isolation, the bed bug was surface sterilized by suspending in 1.0% sodium hypochlorite and subsequently rinsed with autoclaved distilled water thrice^69^. Three biological replicates were randomly selected from each experimental group (control, F0, and F1). In each replicate, three individuals of 7-day-old, unmated adult male bed bug were pooled together to account for variation between individuals of the same treatment. Metagenomic DNA was eluted to a final volume of 50 μL and stored in − 20 °C before being subjected to library preparation and sequencing. The quality and quantity of metagenomic DNA was examined with NanoDrop spectrophotometer (ThermoScientific, Wilmington, DE, USA) and gel electrophoresis. PCR amplification and paired-end DNA sequencing of the V3/V4 regions in the 16S rRNA gene was outsourced to Apical Scientific Sdn Bhd (Selangor, Malaysia) and performed accordingly to their established protocols (see Supplementary Methods).

### Bioinformatics pipeline and analysis

The raw sequencing output from the Illumina 16S rRNA metagenomics sequencing were analyzed using the Quantitative Insights Into Microbial Ecology (QIIME2) v.2020.06 pipeline^70^. Denoising and quality control including barcode and primer sequence removal, quality filtering, correcting errors in marginal sequences, removing chimeric sequences, removing singletons, joining paired end reads, and dereplication were done using the DADA2 plugin within QIIME2^71^. The DADA2 algorithm was also used to cluster representative amplicon sequence variants and provide count frequencies in each sample. Taxonomy classification from phylum to genus was assigned to the representative amplicon sequence variants based on the GreenGenes database v13.5^72^ at 97% operational taxonomic unit (OTU) level, trained using a Naïve Bayes classifier (classify-sklearn) and the feature-classifier QIIME2 plugin. Any sequences affiliated to chloroplast, mitochondria, or without phylum assignation were removed from the OTUs classification. The resulting OTU table were subjected to functional prediction analysis using the software PICRUSt v1.1.1.^41^. The predicted functions were then annotated with the Kyoto Encyclopedia of Genes and Genomes (KEGG) pathways in accordance with their respective functions and KEGG Orthology (KO). The accuracy of the predictions was evaluated based on the Nearest Sequenced Taxon Index (NSTI).

Alpha and beta diversity was determined based on the feature table that was rarefied to a sampling depth of 13,041, which retained 117,369 of sequences in all nine samples. This sampling depth was selected as it was approaching the maximum depth which retained all samples for the diversity analyses^73^. Only OTUs that reached 97% identity level were used for alpha diversity analysis (Shannon’s index, Fisher’s alpha diversity, Pielou’s evenness and Faith’s Phylogeny diversity) using the q2-diversity plugin in QIIME. A webtool Venny v2.1^74^ was used to determine OTUs that are unique or shared between the study groups (Control, F0, and F1). Differences between alpha diversity indices were determined using the Kruskal–Wallis test within QIIME2. Principal coordinate analysis (PCoA) plot based on the Bray–Curtis dissimilarity index were constructed in QIIME2 to compare beta diversity between the groups.

The software STAMP v.2.1.3^75^ was used to provide statistical significance for taxonomic and functional profiles among the study groups based on the relative abundance by using one-way ANOVA and Tukey–Kramer post hoc test, corrected for False Discovery Rate (FDR) using the Benjamini–Hochberg^76^ procedure (α = 0.05). A heatmap was also constructed in STAMP based on the proportion of sequences (%), averaged neighbor UPGMA, and dendogram clustering with a threshold of 0.95 for the top 15 genera across the study groups.

### Statistical analysis

The survivorship of tested insects was described using the restricted mean survival time (RMST) analysis and Kaplan–Meier survival curves were constructed by using the MedCalc Statistical Software version 19.8 (MedCalc Software Ltd, Ostend, Belgium; https://www.medcalc.org). The restricted mean survival time (RMST) is a measure of average survival from the beginning of the study to a specified time point to allow statistical comparison between study groups. In our study, the time point for RMST is determined by the Medcalc statistical software, set to the lowest time point of the last event among the different groups. The statistical significance for fecundity and nymphal development was determined by independent t-test. For enzyme biochemical assays, KT100, and percentage mortality 48 h post-treatment, significance was assessed based on the one-way ANOVA and Duncan’s multiple range test. All statistical analyses were performed using the IBM Statistical Package for Social Sciences (SPSS) v.25 at a significance level of α = 0.05.

### Ethics approval

This study has received the ethics approval from Universiti Sains Malaysia Animal Ethics Committee (USM/Animal Ethics Approval/2016/(104)(819) for bed bugs maintenance.

## Supplementary Information

Supplementary Information 1.

Supplementary Information 2.

## Data Availability

The 16S rRNA sequences determined in this study were deposited in the NCBI Sequence Read Archive (SRA) database (BioProject accession : PRJNA815933). All other information is provided within the manuscript.
