# Supplementary material for: Bacterial symbionts influence host susceptibility to fenitrothion and imidacloprid in the obligate hematophagous bed bug, Cimex hemipterus
Source: Sci Rep. 2022 Mar 22;12:4919. doi: 10.1038/s41598-022-09015-0 (PMC8941108; doi:10.1038/s41598-022-09015-0)
Supplement: Supplementary file 1 — Supplementary Information 1. [file 41598_2022_9015_MOESM1_ESM.docx]

**Bacterial symbionts influence host susceptibility to fenitrothion and imidacloprid in the obligate hematophagous bed bug, *Cimex hemipterus***

Li-Shen Soh^1^ and G. Veera Singham^1^*

*^1^Centre for Chemical Biology, Universiti Sains Malaysia, Bayan Lepas, 11900 Penang, Malaysia.*

* Corresponding author*:* [veerasingham@usm.my](mailto:veerasingham@usm.my)

ORCID: https://orcid.org/0000-0001-9220-370X

**Supplementary Methods**

*General esterase activity*

General esterase activity was determined based on the method of Khalid *et al*. ^67^. The homogenate (10 µL) was mixed with 0.1 M phosphate buffer (40 µL) and 1.0 mM substrate α-naphtyl acetate or β-naphtyl acetate (40 µL). The mixture was then allowed to incubate for 30 minutes at 30 °C. The mixture reaction was terminated by adding in 50 µL of stop solution, which consisted of 0.5 % fast blue RR salt (w/v) and 5 % of sodium dodecyl sulfate (W/V) dissolved in 0.1 M phosphate buffer (pH 7.0). The reaction mixture was left standing for 2 minutes to allow color development (green for substrate α-naphtyl acetate; red for substrate β-naphtyl acetate). The absorbance readings were then taken at 600 nm and 560 nm for α-naphtyl acetate and β- naphtyl acetate, respectively by using a microplate reader Synergy MX (Bio-Tek Instruments, Winooski, VT, USA). α- naphthol or β-naphthol was used to generate the standard curves and the general esterase activity was expressed as μmol/min/mg of protein.

*Glutathione S-transferase (GST) assay*

The quantification of glutathione S-transferase (GST) was determined based on the procedures described by Khalid et al.^67^. The homogenate (10 µL) was mixed with 190 µL of 100 mM phosphate buffer containing 10.5 mM reduced glutathione and 10 µL of methanol containing 63 mM of CDNB (substrate). The absorbance readings were taken at 340 nm every minute for up to 5 minutes by using the microplate reader with path length correction of 0.6 cm. The binding of GST with substrate CDNB resulted in the formation of GST-DNB conjugates. The conjugation increases with time, which correspondingly increases the absorbance readings. GST activity was determined by evaluating the formation rate of the conjugates and expressed in μmol/min/mg.

*Heme peroxidase activity assay*

Heme peroxidase activity was performed using the methods described by Son-un et al.^68^. TMB (6.3 mM) solution was prepared by dissolving 0.01 g of 3,3′,5,5′-Tetramethylbenzidine dihydrochloride hydrate (TMB) (Sigma-Aldrich Co., MO, USA in 5 mL methanol before mixing the solution with 15 mL of 0.25 M sodium acetate buffer (pH 5.0). 200 μL of the TMB solution was then mixed with 25 μL of 3 % hydrogen peroxide (v/v), 20 μL of homogenate and 80 μL of 0.1 M phosphate buffer (pH 7.0). The mixture was incubated at room temperature in the microplate reader for 5 minutes before taking the absorbance reading at 630 nm. The heme peroxidase activity was determined based on the cytochrome C (extracted from bovine heart) standard curve and expressed as μg/mg of protein.

*16S rRNA metagenomics sequencing protocol*

The hypervariable V3/V4 regions of the bacterial 16S rRNA gene were amplified with the locus-specific sequence primers 341F (5’ TCGTCGGCAGCGTCAGATGTGTATAAGAGACAGCCTACGGGNGGCWGCAG-3’) and 805R (5’-GTCTCGTGGGCTCGGAGATGTGTATAAGAGACAGGACTACHVGGGTATCTAATCC-3’), whereby the underlined refers to Illumina overhang adapter sequences. The product was further re-amplified in a limited-cycle PCR reaction to barcode dual indexes into the read. The first PCR reaction was performed in 96-well 0.2 mL PCR plates with 25 μL of reacting solution which comprised of 12.5 μL Q5® High-Fidelity DNA Polymerase (New England Biolabs, MA, USA), 5 μL of each primer (1 μM), and 2.5 μL of purified metagenomic DNA (5 ng/μL). The PCR conditions were programmed as follows: initial denaturation at 95 °C (3 minutes) followed by 25 cycles of the following steps: denaturation at 95 °C for 30 seconds, annealing at 55 °C for 30 seconds, extension at 72 °C for 30 seconds. The PCR cycle was terminated with a final extension at 72 °C for 5 min. In the second PCR, indexes were added to both ends of the amplified target region using the Nextera XT Index Kit v2 (Illumina, USA) following the manufacturer’s protocols. The quality of the libraries was evaluated using the Agilent Bioanalyzer 2100 System by Agilent DNA 1000 Kit (Agilent Technologies, Germany) and fluorometric quantification by Helixyte GreenTM Quantifying Reagent (AAT Bioquest, Inc., CA). The libraries were normalized and pooled according to the protocol recommended by Illumina and subjected to MiSeq platform (Illumina, USA) with 300 PE.

**Supplementary Table S1** 16S rRNA sequence processing data

| Study Group | Sample ID | Raw reads | Filtered reads | Denoised reads | Merged reads | Non-chimeric reads | Final reads |
| --- | --- | --- | --- | --- | --- | --- | --- |
| Control | C1 | 212,797 | 151,182 | 150,758 | 150,110 | 29,780 | 29,709 |
|  | C2 | 318,936 | 240,484 | 239,935 | 239,196 | 41,998 | 41,953 |
|  | C3 | 319,948 | 184,771 | 184,263 | 183,776 | 27,805 | 27,794 |
| F0 | S1 | 259,336 | 186,981 | 186,504 | 186,028 | 43,610 | 43,560 |
|  | S2 | 454,703 | 235,303 | 234,725 | 233,878 | 43,710 | 43,696 |
|  | S3 | 442,957 | 209,503 | 208,901 | 207,847 | 40,564 | 40,552 |
| F1 | L1 | 232,015 | 142,075 | 141,105 | 137,349 | 16,312 | 16,287 |
|  | L2 | 262,430 | 168,301 | 167,020 | 163,105 | 16,154 | 15,115 |
|  | L3 | 442,823 | 121,557 | 120,675 | 117,292 | 13,128 | 13,041 |
|  | Total | 2,945,945 | 1,640,157 | 1,633,886 | 1,618,581 | 273,061 | 271,707 |


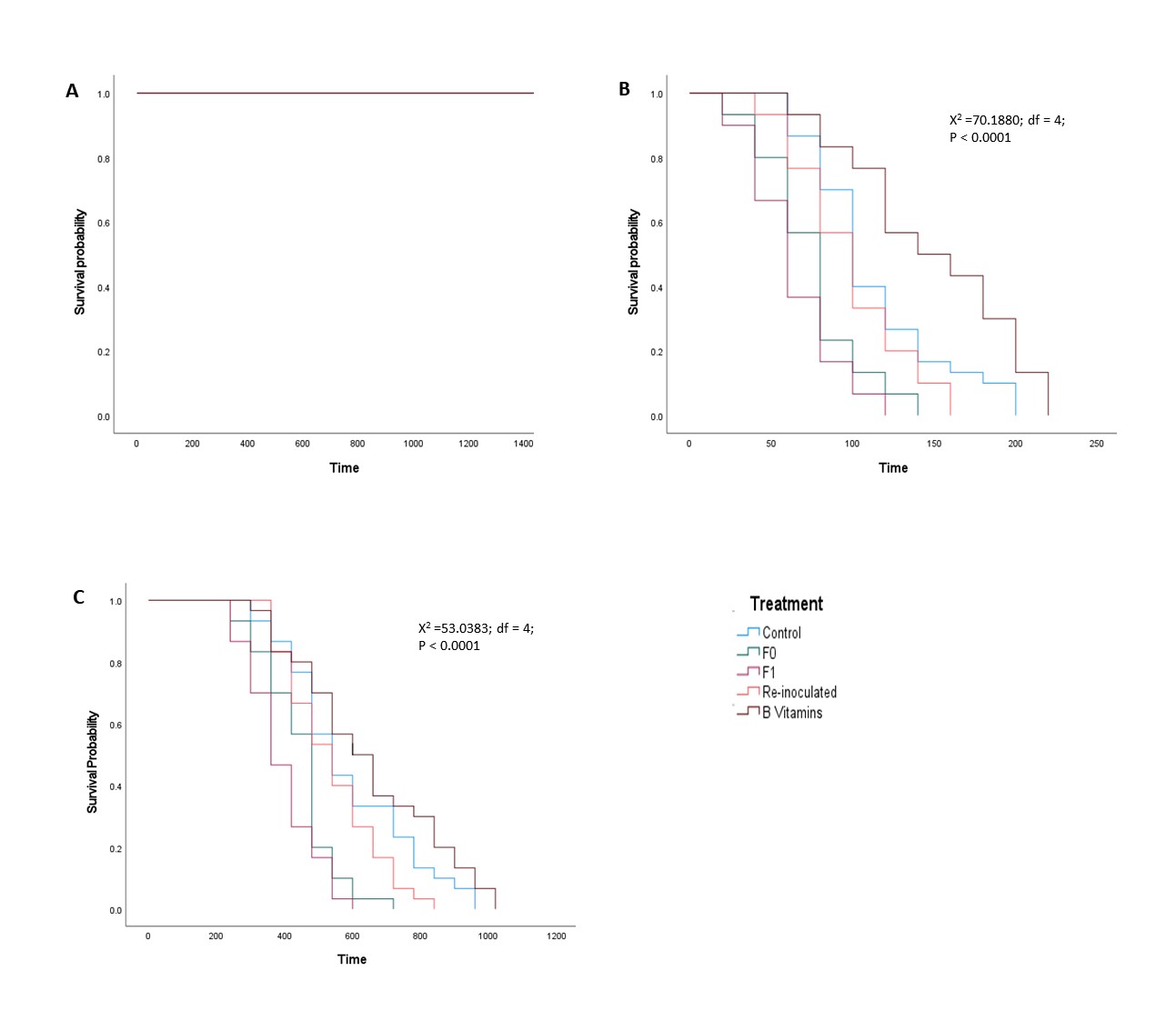


**Supplementary Fig. S1**. Kaplan-Meier survival analysis of *C. hemipterus* under different test conditions (control, F0, F1, re-inoculated, and B Vitamins) when subjected to different insecticides. (a) deltamethrin; (b) imidacloprid; (c) fenitrothion.


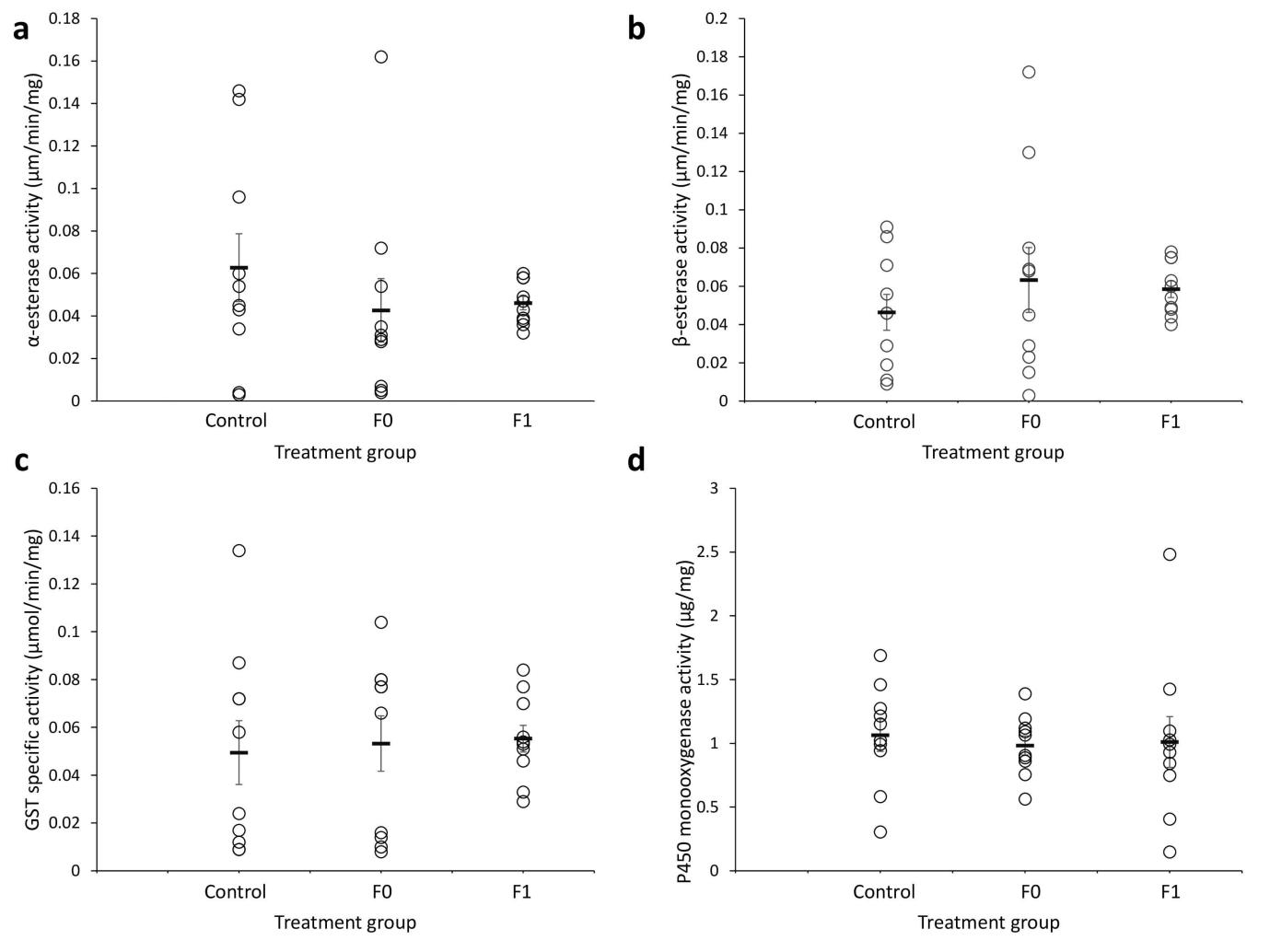


**Supplementary Fig. S2**. Comparison of metabolic enzyme activities between antibiotic treated (F0 and F1) and control *C. hemipterus*. (a) α-esterase activity; (b) β-esterase activity; (c) GST specific activity; (d) P450 monooxygenase activity. N=10 for each study groups. No significant differences were observed among the different experimental groups (control, F0 and F1) across all enzyme assays (One-way ANOVA and Duncan’s multiple range test; *P*>0.05).
